# Imaging Software-Based Sarcopenia Assessment in Gastroenterology: Evolution and Clinical Meaning

**DOI:** 10.1155/2021/6669480

**Published:** 2021-01-04

**Authors:** Giovanni Marasco, Sinan Sadalla, Giulio Vara, Rita Golfieri, Davide Festi, Antonio Colecchia, Matteo Renzulli

**Affiliations:** ^1^Division of Gastroenterology, IRCCS, Azienda Ospedaliero-Universitaria di Bologna, Bologna, Italy; ^2^Department of Medical and Surgical Sciences, University of Bologna, Via Massarenti 9, Bologna 40138, Italy; ^3^Division of Radiology, IRCCS Azienda Ospedaliero-Universitaria di Bologna, Bologna, Italy; ^4^Unit of Gastroenterology, Borgo Trento University Hospital of Verona, Verona, Italy

## Abstract

Sarcopenia is gaining attention as a negative prognostic factor in different fields of medicine, including chronic liver failure. However, the assessment of sarcopenia in patients with liver diseases is often neglected due to unawareness of reliable tools and methods and thus is limited to research studies. Cross-sectional imaging is a diffuse diagnostic tool and is commonly performed in patients with chronic liver failure. The last advancements in radiology image analysis using dedicated software allow an easy and standardized method to assess skeletal muscle volume. Several measures can be obtained from cross-sectional imaging analysis to evaluate sarcopenia in patients affected by chronic liver disease. We aimed to review the recent advances in imaging-based sarcopenia assessment, in particular in patients with chronic liver diseases. As a result, we found that the skeletal muscle index (SMI) seems to be a reliable method to assess sarcopenia in cirrhotic patients. Even if further studies are needed to validate proper cut-offs for each clinical endpoint, physicians are invited to consider the assessment of sarcopenia in the work-up of patients with chronic liver disease.

## 1. Introduction

**Figure 1:**
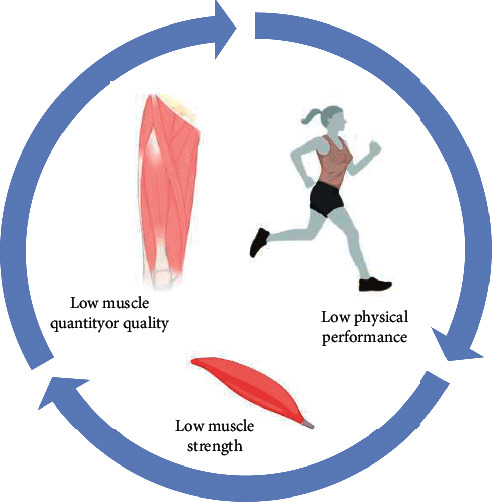
Sarcopenia impact on physical performance.

Sarcopenia, firstly described as age-related loss of muscle mass [1], is now recognized as a skeletal muscle disorder characterized by a loss of muscle strength with a concomitant loss of muscle mass and function [2, 3]. While sarcopenia was previously considered a normal consequence of aging, in the last decade new evidence showed that it can also develop in adults [3]. Sarcopenia is characterized by multifactorial pathogenesis and results from a mismatch between protein synthesis and breakdown [4] (Figure 1).

In the last decade, various scientific associations attempted to develop operational definitions of sarcopenia. In 2014, the Foundation for the National Institute of Health Sarcopenia Project [5] identified clinically relevant cut-points for muscle grip, mobility impairment [6], and low muscle mass [7]. In 2019, the European Working Group on Sarcopenia in Older People (EWGSOP2) [3] updated their previous operative definition of sarcopenia and identified low muscle strength as the stronger predictor of adverse outcomes; low muscle quantity or quality and low physical performance were considered secondary criteria. Moreover, sarcopenia is associated with recurrent falls and fractures [8] and is predictive of poor outcome in different clinical settings, from oncology [9] to the intensive care unit [10]. Recent evidence focused on the prognostic role of sarcopenia in patients with liver disease [11, 12]. Given the consistent prevalence of sarcopenia in cirrhotic patients (10% in Child A, 34% in Child B, and 54% in Child C patients) [13], early recognition of skeletal muscle changes in these patients is demanded.

Patients with chronic liver diseases often become malnourished due to increased energy expenditure, anorexia, alterations in circulating levels of hormones [14], altered nutrient metabolism, and malabsorption [15], all factors that contribute to muscle atrophy. However, objective nutrition status analysis in cirrhotic patients suffers the biases of retained fluid, ascites, and altered protein synthesis [16]. Even if malnutrition and sarcopenia are different conditions, they often overlap and the latter is now considered a diagnostic criterion of the former [17]. Recent evidence showed that sarcopenia in cirrhotic patients is an independent predictor of lower survival and is associated with an increased hospital stay [18], a higher rate of complications [19, 20], poorer outcomes in patients with hepatocellular carcinoma (HCC) [21], and worse quality of life [22]. It has also been associated with increased waiting list mortality [13] and is a predictor of a higher rate of infections in patients following liver transplantation [23]. Furthermore, a MELD-sarcopenia score has been proposed as a more precise tool to predict mortality in cirrhotic patients [24]. There are different methods to assess the severity of liver diseases, the most diffuse being the Child–Turcotte–Pugh (CPT) score and the model for end-stage liver disease (MELD). These scores are used to predict mortality in cirrhotic patients [25] and, in addition, MELD is used in liver transplant allocation list [26, 27]. These scores are composed of clinical and laboratory parameters but do not consider the nutritional status of patients [18]. In this regard, an accurate assessment of sarcopenia could allow an objective and easy measurement of nutrition status in cirrhotic patients [18].

## 2. Methods

This narrative review aims to describe the recent advances in sarcopenia assessment in patients with chronic liver diseases, focusing on the technical side of this practice. We conducted a PubMed, MEDLINE, and Scopus search from inception to August 2020 using the search terms “sarcopenia,” “chronic liver disease,” “cirrhosis,” “computed tomography,” and “malnutrition” followed by a manual review of the literature to select relevant articles for this clinical review. Literature research was carried out based on title and abstract without time and language restrictions. Article search was carried out independently by two authors (GM and SS).

## 3. Results

### 3.1. Methods for Assessing Sarcopenia

There are different clinical tools to detect sarcopenia [28], from anthropometrical measures (i.e., mid-arm muscular circumference, triceps skinfold thickness) to bioelectrical impendence analysis, hand-grip strength, and the chair stand test. However, radiologic exams like whole-body dual-energy x-ray absorptiometry (DEXA), magnetic resonance imaging (MRI), and computed tomography (CT) are generally considered the “gold standard” to assess body composition [29].

**Figure 2:**
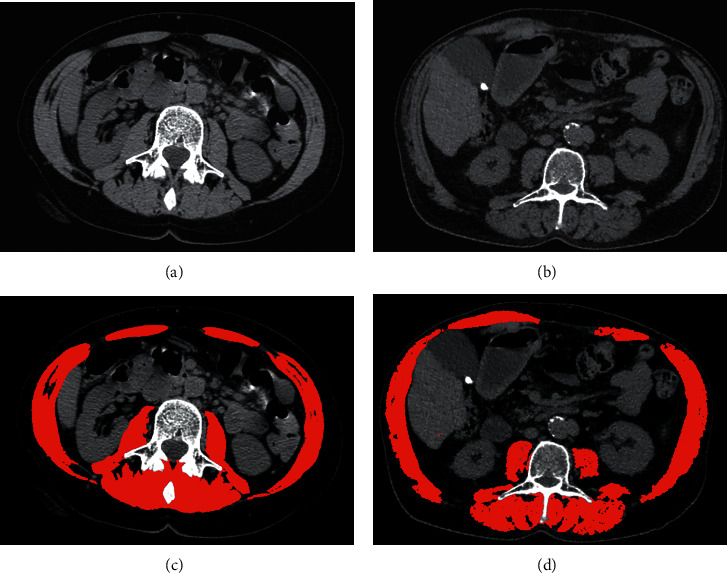
Computed tomography images of two different patients, one without cirrhosis (a) and one with cirrhosis (b). The patient without cirrhosis does not demonstrate imaging sign of sarcopenia using the public domain software (ImageJ) (c). The patient with cirrhosis demonstrates imaging sign of sarcopenia using the public domain software (ImageJ) (d).

CT image analysis is a diffuse imaging technique and a precise method for the evaluation of human body composition [29] (Figure 2). CT scan provides the capacity to discern between different body tissues (adipose, skeletal muscle, bone, water, and air) based on tissue-specific attenuation values and thresholds, measured in Hounsfield Units (HU). Recent evidence highlighted the diagnostic and prognostic role of sarcopenia assessment by CT scan in cirrhotic patients [30]. Using specific software, CT scan allows an easy measure of skeletal muscle area (SMA), which is then adjusted for height to obtain the skeletal muscle index (SMI, cm^2^/m^2^) [28].

From the introduction of the first computed tomography (CT) in the last years of 1960s, the radiologic field underwent a constant evolution [31]. In 1978, Abrams and McNeil described the use of CT imaging for quantifying anatomic detail [32]. They demonstrated that using tissue-specific threshold values CT was able to distinguish the different tissues of the body.

In the last decades, cross-sectional imaging analysis allowed performing volumetric studies and determining tissue quantification, such as adipose and muscle tissue. In the first period, body compartments volumes were calculated using whole-body cross-sectional imaging (CT scan and MRI). Researchers had to quantify tissue areas for each slice, and then through the use of geometrical models, it was possible to estimate the volumes of different compartments [33]. However, this method was time-consuming and required whole-body imaging. Moreover, while total body imaging is often performed in a research context, the most frequent imaging procedures in clinical practice are focused on the abdomen region.

In an attempt to find an easier way to obtain a measure of body compartments, Shen et al. [34] demonstrated in 2004 that single abdominal measures of muscle and adipose tissues obtained with cross-sectional MRI scans were highly correlated with the respective total-body volumes. The authors found that the highest correlation between a single slice of skeletal muscle and total body muscle volume was at 5 centimeters above the L4-L5 level.

In fact, the abdominal region, from L3 to the iliac crest, contains several muscle groups: the psoas, paraspinal muscles (erector spinae and quadratus lumborum), and abdominal wall muscles (transversus abdominus, external and internal obliques, and rectus abdominus). Moreover, while appendicular muscles are influenced by activity level, the abdominal muscle mass is relatively independent of physical exercise [35]. This result, obtained in a large sample of healthy adults, made the measurement of total skeletal muscle volume affordable and immediate and therefore more usable in daily clinical practice.

Therefore, given the correlation between a single measure and whole-body compartments, total body muscle mass can be easily assessed from a single measure, such as the L3-L4 skeletal muscle index, the psoas muscle area, and the dorsal muscle area.

### 3.2. Imaging Software for Sarcopenia Evaluation

Different image analysis methods and tools can be used to measure sarcopenia, most of which are based on automatic and semi-automatic software. Radiologic image analysis is the most diffuse technique to assess sarcopenia in patients with chronic liver disease, and these can be done in different ways [36]. The major studies that focused on cross-sectional imaging-derived sarcopenia and its prognostic role [9, 29] chose L3 as the standard landmark and used two consecutive images from L3 to iliac crest to measure muscle cross-sectional area. CT HU threshold to detect skeletal muscle ranged from −29 to +150, as previously identified by Mitsiopoulos et al. [37]. The majority of studies [9, 12, 29, 38, 39] used specific automatic software (the Slice-O-Matic software, version 4.3 Tomovision, Montreal, QC, Canada; Mimics software version 14, Materialise, Leuven, Belgium) to analyse the images. Muscles cross-sectional areas (cm^2^) were evaluated on two consecutive CT slices at L3 level. The software analysed the different areas based on HU range and manual delineation of tissue boundaries was performed when necessary. The final skeletal muscle area was the mean of the measures derived from the two different levels. Given the existence of large databases of CT scan that could be used for retrospective analysis, the main limit for the application of these results is the costs of the proprietary software. As a result, many authors searched for different methods to overcome this limitation [16, 40]. At the state of the art, different methods are available to assess skeletal muscle surface in radiology imaging without additional costs, alternative to the use of proprietary software. Among these, two techniques demand attention: the first method is an open-source software for image processing [40] while the second uses a linear measurement that previous studies identified as a surrogate of sarcopenia [16]. In particular, Gomez-Perez et al. [40] described in their tutorial how to use the free public domain software developed by the National Institutes of Health (NIH-ImageJ). Starting from a single cross-sectional image at the midpoint of the lumbar vertebrae L3, the software requires manual tracing of the various abdominal regions to obtain body composition information. The first thing to do is to delimit the abdominal perimeter, as it is a valid surrogate for the standing waist circumference. Second, the operator must draw the outside and inside perimeters of the abdominal muscles included in the image. Using these results and a preprogrammed template, the operator can easily determine waist circumference and skeletal muscle area that, combined with the squared height of the patient, will generate the skeletal muscle index (SMI, cm^2^/m^2^) (Figure 2).

### 3.3. The Clinical Meaning of Sarcopenia in Solid Tumors

**Figure 3:**
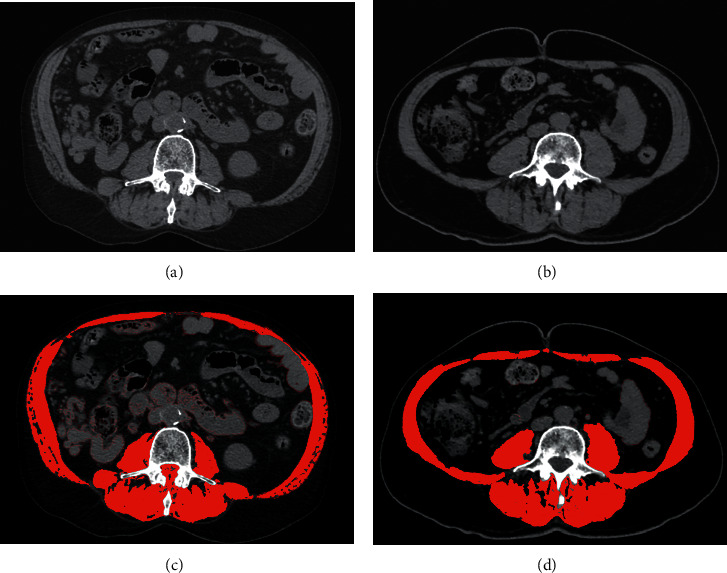
Computed tomography images of two different patients, one affected by HCC (a) and one affected by pancreatic cancer (b). Both patients demonstrate imaging sign of sarcopenia using the public domain software (ImageJ) (B B').

The skeletal muscle index assessed at L3 level is the most diffuse technique to assess sarcopenia in patients with solid tumors and cirrhosis [41] (Figure 3). As example, Mourtzarkis et al. [29] showed that, in a group of oncological patients including those with gastrointestinal and liver tumors, CT scan analysis at the third lumbar vertebrae (L3) level was a stronger predictor of total body fat-free mass compared to other radiological analysis such as DEXA and bioelectrical impedance analysis (BIA). The authors used two consecutive abdominal images at L3 level to develop and validate regression equations to predict whole-body composition of adipose tissue and skeletal muscle mass in patients with cancer. Given the previous results, other studies focused on the prognostic role of imaging-derived sarcopenia. Prado et al. [9] evaluated the clinical implications of sarcopenia in obese patients with gastrointestinal and respiratory solid tumors; these authors used CT scans to calculate the L3 skeletal muscle index (skeletal muscle area adjusted for height), which was correlated with whole-body skeletal muscle mass. Thereafter, they [9] determined sex-specific cut-points for sarcopenia (males ≤52.4 cm^2^/m^2^, females ≤38.5 cm^2^/m^2^) that were associated with increased mortality. Following these results, different studies applied these cut-offs in different populations, showing a higher association with adverse outcomes in intensive care unit (ICU) patients [38, 39, 42] and in oncological patients [43, 44].

### 3.4. The Clinical Meaning of Sarcopenia in Liver Diseases

Moreover, this method to assess sarcopenia was evaluated in patients with liver disease and HCC [12, 21, 45, 46]. As abovementioned, cross imaging analysis allows easy quantification of sarcopenia in cirrhotic patients. However, the measures and cut-points used in the previous studies [9, 29] were derived from an obese population of oncological and non-cirrhotic patients. Even if a recent study based on clinical outcomes suggested specific cut-offs (50 cm^2^/m^2^ for men and 39 cm^2^/m^2^ for women) [30], cut-points for sarcopenia in cirrhotic patients still lack consensus. Moreover, in a recent meta-analysis, Kim et al. [18] assessed the role of ethnicity impact on sarcopenia in patients suffering from liver cirrhosis; compared to the Western population, sarcopenia in the Eastern population was associated with higher mortality. Therefore, the assessment of skeletal muscle mass in liver disease still needs further validation studies [47]. In 2017, Carey et al. [30] conducted a large multicenter study to establish sex-specific cut-off values of SMI in patients at end-stage liver disease awaiting liver transplantation. However, manual delineation of different muscle groups is relatively complex, takes some time, and is difficult to standardize. Therefore, different CT-derived measures have been proposed, such as paraspinal muscle index (PSMI) and total psoas muscle volume (TPV) [48]. Durand et al. [16] proposed linear measure of psoas muscle on CT at the level of the umbilicus in a population of patients with cirrhosis on the waiting list for liver transplantation performed in predicting mortality. Psoas muscle was selected among other body markers because it can be easily identified on a CT and because, compared with parietal muscles, it is not directly affected by abdominal distension in patients with ascites. The authors used pretransplant CT to obtain axial and transverse thickness measures of the right psoas muscle, which was normalized by dividing with the patient height (PMTH). As a result, PMTH was predictive of waiting list mortality independently of the MELD score with an optimal cut-point of 16.8 mm/m. Gu et al. [41] used this value and previously published cut-off [12] to determine both PMTH and SMI in patients with liver cirrhosis. PMTH, simpler to be calculated compared to other indexes and not requiring software, was found to be correlated with SMI and an independent prognostic factor for mortality in cirrhotic patients. Moreover, sex-specific PMTH-based sarcopenia was significantly associated with mortality, while sex non-specific PMTH was not correlated with mortality. However, this method carries some weak points, such as a variable landmark (the umbilicus) to measure muscle thickness; thus, a single muscle measure still cannot be recommended [28]. In conclusion, sarcopenia in cirrhotic patients is associated with poorer outcomes. A large proportion of cirrhotic patients has a CT scan performed in their medical history for HCC surveillance and/or liver transplant evaluation, so that deriving skeletal mass would be useful to adopt strategies to maintain or improve muscle mass. Cut-points values to assess sarcopenia in cirrhotic patients have recently been proposed but need further validation [30]. Given the presence of open-source software, sarcopenia detection can be rapid and cost-free. Among the different measures to determine sarcopenia from cross-sectional imaging, skeletal muscle index (SMI) at L3 level is diffuse and has been validated in different settings; thus, it should be recommended [49]. Notably, previous research found that, in patients waiting for liver transplantation, a subgroup at high risk for early mortality may not be appropriately ranked by the existing MELD score [16]. Moreover, sarcopenia appears to have a prognostic value in the liver transplantation setting [13].

## 4. Conclusions

CT detection of sarcopenia is easy to perform and, if future studies will validate these data, it will be used in the daily clinical practice in almost all the hospitals where CT is available. Routine evaluation of body mass composition should be adopted to detect sarcopenia in cirrhotic patients in order to improve an adequate stratification of mortality risk.

## Data Availability

No data were used to support this study.
